# Cryoproteins in Non-HCV-Related Autoimmune Disorders: A Serious Cold-Induced Problem

**DOI:** 10.3390/diagnostics15151933

**Published:** 2025-07-31

**Authors:** Krizia Pocino, Annunziata Stefanile, Patrizia Natali, Cecilia Napodano, Valerio Basile, Gabriele Ciasca, Mariapaola Marino, Umberto Basile

**Affiliations:** 1 Unità Operativa Complessa di Patologia Clinica, Ospedale San Pietro Fatebenefratelli, 00189 Rome, Italy; pocino.krizia@fbfrm.it (K.P.); stefanile.nunzia@gmail.com (A.S.); 2 Dipartimento Interaziendale Integrato di Medicina di Laboratorio, Azienda Unità Sanitaria Locale e Azienda Ospedaliero-Universitaria di Modena, 41125 Modena, Italy; p.natali@ausl.mo.it (P.N.); cecilia.napodano@gmail.com (C.N.); 3 Clinical Pathology Unit and Cancer Biobank, Department of Research and Advanced Technologies, Regina Elena National Cancer Institute IRCCS, 00144 Rome, Italy; valeriobasile90@gmail.com; 4 Fondazione Policlinico Universitario “A. Gemelli” I.R.C.C.S., 00168 Rome, Italy; gabriele.ciasca@unicatt.it; 5 Sezione di Fisica, Dipartimento di Neuroscienze, Università Cattolica del Sacro, 00168 Rome, Italy; 6 Sezione di Patologia Generale, Dipartimento di Medicina e Chirurgia Traslazionale, Università Cattolica del Sacro Cuore, 00168 Rome, Italy; 7 Department of Clinical Pathology, Santa Maria Goretti Hospital, 04100 Latina, Italy; u.basile@ausl.latina.it

**Keywords:** cryoglobulins, cryocrit, cryofibrinogen, cold agglutinins

## Abstract

The precipitation of cryoglobulins, serum immunoglobulins, below 37 °C defines the clinical cryoglobulinemic syndrome, a systemic vasculitis usually characterized by purpura, weakness, and arthralgia. In most cases, this condition is associated with chronic infection by the hepatitis C virus (HCV) and can evolve into B-cell dysregulation and malignancies. The current literature on non-HCV-associated cryoglobulinemia is very limited, and little is known about the immunological and serological profile of affected patients. The cryoglobulinemic syndrome not associated with HCV infection is often found concomitantly with other infections, autoimmune diseases, and B-cell lymphoproliferative disorders. The cryoprecipitation of fibrinogen has been described as a rare disorder, perhaps underestimated and not fully understood, causing thrombotic occlusion and ischemia in different rheumatic disorders. Cold temperature plays a pathogenetic role in autoimmune hemolytic anemias, in which the presence of cold agglutinins produced by B cells at the lymphoplasmacytic cell stage may promote agglutination of red blood cells in the coldest parts of the circulation, even at mild room temperatures, undergoing hemolysis. Laboratory methods for the detection and quantification of cryoproteins are downright critical, and their concurrent detection is pivotal for the diagnosis. In this review, we summarize the clinical involvement of cryoglobulins, cryofibrinogen, and cold agglutinins in non-HCV autoimmune diseases, underlining the crucial steps of the most employed analytic methods.

## 1. Cryoglobulins and Autoimmunity

Cryoglobulins (CGs) comprise proteins circulating in human serum that precipitate in vitro at temperatures lower than 37 °C and re-solubilize upon warming [1,2]. The cold-induced precipitation of serum proteins was first described in 1933 by Wintrobe and Buell [1], and Lerner and Watson introduced the term cryoglobulinemia in 1947 [2]. In 1966, Meltzer and Franklin described the typical clinical symptoms associated with cryoglobulinemia (the Meltzer triad: purpura, arthralgia, and weakness) [3].

Mixed cryoglobulinemia (MC) is a multifactorial disease characterized by the presence of circulating cryoprecipitable immune complexes in the serum; HCV appears to have a preeminent etiologic role in MC, since HCV infection can be found in 76–95% of patients with MC [4]. These cryoglobulins can consist of immune complexes containing rheumatoid factor (RF), known as “mixed” CGs to differentiate them from monoclonal CGs, which do not contain RF or antigen–antibody complexes [5].

Cryoglobulins maintain their solubility even when aggregated in optimal thermal conditions. A reduction in temperature provokes steric modifications of molecules with exposition of non-polar residua, reduction in solubility and cryoprecipitation; it probably occurs because of the rapid formation of cold-insoluble IgM-IgG (immunoglobulin type M/G) immune complexes or simply by a decreasing solubility resulting from an unfavorable interaction between CGs and solvent at low temperatures [6]. When the temperature rewarms at 37 °C, molecules return to the initial conformation [5].

This event represents the pathogenic mechanism of a wide range of symptoms. They deposit in small- to medium-sized blood vessels throughout the body, causing endothelial injury and end-organ damage known as cryoglobulinemia [7,8]. Organs commonly affected include the skin, kidneys, and peripheral nerves, with clinical manifestations such as purpura, glomerulonephritis, and neuropathy [7]. Based on the characteristics of the involved immunoglobulins, CGs have been classified by Brouet et al. as type I if they are formed of a single monoclonal immunoglobulin; type II if they are formed of a monoclonal immunoglobulin and polyclonal immunoglobulin; and type III if only polyclonal immunoglobulins are found [9] (Table 1). The key difference between type II and type III CGs lies in whether RF is monoclonal or polyclonal. Both types involve autoantibodies (Auto-Abs) and may contain monoclonal RF IgM and/or IgG [10]. Type I CGs are typically associated with lymphoproliferative disorders and can present with symptoms such as vasculitis or distal gangrene/necrosis. In contrast, type II and III CGs are found in a broad range of conditions, including infectious diseases (especially HCV infection), autoimmune disorders, lymphoproliferative diseases, and chronic liver diseases. These types commonly manifest with symptoms like purpura, joint pain (arthralgia), and Raynaud’s phenomenon [11] (Table 1).

The current literature on non-HCV-associated cryoglobulinemia is very limited, and little is known about the immunological and serological profile of affected patients [12]. Saadoun et al. described clinical signs and outcomes of a group of patients with non-HCV-related mixed cryoglobulinemia. The immunological features consisted of low C3 and C4 complement levels, RF activity, antinuclear antibodies (ANA), anti-Sjögren’s syndrome type A (anti-SSA) and anticardiolipin antibodies with different frequencies [13].

In most cases, when a patient with cryoglobulinemic vasculitis also presents with liver disease, it is commonly linked to HCV infection. However, a recent case report highlights an unusual instance involving a patient diagnosed with both cryoglobulinemic vasculitis and autoimmune hepatitis [14]. Autoimmune hepatitis is a rare, immune-mediated inflammatory liver condition, characterized by circulating autoantibodies, elevated IgG levels, and specific histopathological features [15]. Although Meltzer’s triad is a recognized clinical feature of mixed cryoglobulinemia, it appears in only about one-third of cases [16]; notably, this particular patient did not exhibit the triad of purpura, arthralgia, and weakness but presented urticaria on bilateral lower limbs, mild hepatomegaly, anemia, and altered liver function tests; HBV and HCV serology were negative, while ANA, P-ANCA, and serum cryoglobulins were positive, and skin biopsy revealed leukocytoclastic vasculitis. Liver biopsy confirmed the diagnosis of autoimmune hepatitis. The patient was successfully treated with prednisolone and azathioprine [14].

The literature documents three additional cases that highlight the association between cryoglobulinemic vasculitis and autoimmune hepatitis. One notable case involved a 73-year-old man who presented with palpable purpura on his lower limbs and a marked elevation in aminotransferases—closely mirroring the clinical presentation described in a previous report [17]. Another intriguing case, described by Biecker et al., reported the coexistence of celiac disease, autoimmune hepatitis, and cryoglobulinemic vasculitis [18]. The patient exhibited type II cryoglobulinemia that was negative for hepatitis C virus (HCV), along with elevated liver enzymes, iron deficiency, positive antinuclear antibodies (ANA), and increased IgG levels. A liver biopsy confirmed type I autoimmune hepatitis with a possible overlap with autoimmune cholangitis. Treatment with immunosuppressive therapy led to normalization of transaminase levels and resolution of the cryoglobulinemic vasculitis. While celiac disease is a known trigger for autoimmune conditions and is commonly associated with other autoimmune disorders, its connection to autoimmune hepatitis or cholangitis is rarely reported [18].

Additionally, Evans et al. described a case involving autoimmune hepatitis and cryoglobulinemia in which the vasculitis progressed to renal involvement, resulting in glomerulonephritis and anuric acute kidney failure [19]. The patient presented with laboratory findings indicative of liver failure due to acute hepatic necrosis. Viral hepatitis serologies were negative; however, ANA was positive at a titer of 1:320 in a nucleolar pattern. Serum protein electrophoresis revealed elevated gamma globulin levels, and coagulation tests were abnormal. Liver biopsy confirms the suspicion of autoimmune hepatitis. Oral prednisone was started with a significant improvement of symptoms. After a few weeks, the patient showed mild renal insufficiency and elevated RF levels. With a rapid renal deterioration, the patient was placed on dialysis. A transjugular renal biopsy showed a cryoglobulinemic nephritis. Detected serum cryoglobulins were polyclonal. There were no other vasculitis manifestations of cryoglobulinemia. Therapy with Rituximab was immediately effective, and the patient resolved the renal injury. This case report suggests that anti-B cell therapy with rituximab is a reasonable option when cryoglobulinemic glomerulonephritis occurs [19,20].

Sjögren’s disease is a classic and heterogeneous autoimmune disorder primarily characterized by dysfunction of the exocrine glands [21]. Approximately 10% of patients develop severe extraglandular complications, such as cryoglobulinemia, which contribute significantly to the increased morbidity and mortality associated with the disease [22]. The main autoimmune mechanisms involve two key phenomena: first, lymphocytic infiltration of the exocrine glands, mainly composed of autoreactive CD4+ T cells and B cells; second, polyclonal hyperactivity of autoreactive B cells, which leads to the formation of germinal centers in 20–25% of patients and results in heightened production of autoantibodies targeting immunoglobulins, cellular, nuclear, and other antigens, alongside activation of type-I interferon pathways [23,24]. B cells play a central role in the disease process, as they clonally expand and produce monoclonal IgM RF that forms cold-precipitable immune complexes responsible for vasculitis. These clonal B cells exhibit distinctive features, including low CD21 expression and signs of functional exhaustion [25,26].

In Sjögren’s disease, cryoglobulin production is thought to be driven by chronic antigenic stimulation, possibly from an unidentified viral antigen or an autoantigen, with the inflamed salivary epithelium serving as the site of cryoglobulin synthesis [27]. Salivary gland epithelial cells contribute to the autoimmune response by facilitating the presentation of Ro/SSA and La/SSB autoantigens to the immune system. This occurs through increased apoptosis and release of autoantigens within apoptotic bodies, or via secretion of autoantigen-containing exosomes. The expression of these autoantigens in epithelial cells is tightly regulated. Current evidence suggests that signaling through innate immunity receptors like TLR3 influences Ro/SSA and La/SSB expression in salivary gland epithelial cells. Additionally, dysregulated expression of certain microRNAs predicted to target these autoantigens in Sjögren’s patients indicates a post-transcriptional regulatory feedback mechanism [28,29,30].

Most patients with cryoglobulinemic vasculitis associated with Sjögren’s syndrome report fatigue (80–90%), but the most common initial symptom of this vasculitis is palpable purpura (70–90%), typically appearing on the lower limbs and often leaving behind a brownish discoloration as it fades. Other possible skin manifestations include large ulcers above the malleoli, digital necrosis, and livedo racemose [23]. The most frequent neurological complication, affecting 60–70% of patients, is a distal painful sensory or sensorimotor polyneuropathy caused by vasculitis of the vasa nervorum. Additionally, renal involvement is seen in 20–35% of these patients [31].

A large study involving 12,753 individuals with Sjögren’s syndrome revealed that the immunological profile largely depends on the age at diagnosis. Specifically, there is a notable and sustained decline in the positivity rates for the four main Sjögren’s-related autoantibodies—ANA, Ro, La, and RF—with increasing age at diagnosis [32]. Interestingly, ANA positivity decreases gradually until about age 65, after which it begins to rise again, possibly reflecting the generally higher ANA prevalence in older adults. The age-related patterns for Ro, La, and RF antibodies follow a similar trend. This finding was supported by Theander et al., who reported that patients diagnosed before age 40 exhibited the highest frequencies of positive autoantibodies (including ANA, RF, Ro 60/SSA, Ro 52/SSA, and La/SSB), along with higher antibody titers and a broader range of autoantibody specificities within the same samples [33].

It seems that some SS patients are likely to be diagnosed earlier due to the development of systemic disease before glandular dysfunction becomes clinically apparent. Patients carrying RF, anti-Ro and anti-La are diagnosed at a younger mean age, and anti-Ro/SS-A and anti-La/SS-B antibodies are closely associated with global systemic activity.

Cryoglobulinaemia follows a more oscillating pattern, with a tendency to increase with age [32].

The role of cryoglobulins in the pathogenesis of rheumatic diseases is being widely investigated, including systemic lupus erythematosus (SLE).

The most frequently found autoantibodies in cryoprecipitates of patients with SLE were anti-dsDNA, anti-single-stranded DNA, and, less frequently present, antiribonucleoprotein [34]. These autoantibodies are more concentrated in cryoprecipitates than in serum and are correlated with the autoantibodies found in the elution of glomeruli of patients with lupus nephritis [35,36].

Patients with Raynaud’s syndrome exhibit significantly higher levels of cryoprecipitating IgM RF compared to other patients. Additionally, there is a correlation between IgG anti-poly(A) antibody levels within cryoglobulins and disease activity. However, no differences have been found in the composition of cryoglobulins between patients with nephritis and those without obvious renal involvement. Therefore, while the presence of cryoglobulins in SLE indicates active disease, it does not necessarily signify renal involvement [37].

Adu and Williams demonstrated that cryoglobulins from SLE patients can activate complement in vitro, implying that these immune complexes may also trigger complement activation in vivo and contribute to tissue damage in the disease [38].

Lymphocytotoxic antibodies are present in the sera of most SLE patients. The levels of lymphocytotoxic activity in cryoprecipitates generally reflect the corresponding serum titers. However, when adjusted for IgM concentration, it becomes clear that lymphocytotoxic antibodies are selectively concentrated in cryoglobulins, identifying them as IgM antibodies. Despite this, the lymphocytotoxic activity of cryoglobulins does not correlate with SLE severity. Their titers are independent of the presence of active lupus nephritis, total protein or immunoglobulin content in cryoprecipitates, serum complement levels, or circulating anti-DNA antibodies [39].

Thus, although cryoprecipitable lymphocytotoxic antibodies are of theoretical interest, they do not appear to correspond to clinically significant manifestations in SLE.

An association between systemic sclerosis (SSc) and cryoglobulins has been reported. However, due to its rarity, the clinical, biological, morphological, and prognostic implications of this combination remain unclear, and its true prevalence may be underestimated [40,41,42].

SSc is a connective tissue disorder characterized by excessive fibrosis and microvascular damage of the skin and various internal organs [43]. Systemic autoimmunity is one of the central features of the disease. ANAs are detected in more than 90% of patients with this pathology. The two main and specific subtypes are found to be exclusive; anti-Scl-70 (also called anti-topoisomerase I) antibodies seem to be more frequent in patients with diffuse cutaneous SSc, interstitial lung disease, or scleroderma renal crisis. Anti-centromere antibodies are more frequent in patients with limited cutaneous SSc [44].

Patients who test positive for anti-RNA polymerase III (anti-RNAP) antibodies have been reported to face a higher risk of diffuse cutaneous involvement, scleroderma renal crisis, and cancer [45,46]. Deguchi A. et al. described a case of systemic sclerosis (SSc) with anti-RNAP III antibodies complicated by digital gangrene triggered by cryoglobulinemia [47].

It is plausible that the coexistence of systemic autoimmune disorders such as SSc and mixed cryoglobulinemia represents a complex immune-mediated condition marked by particularly severe vascular complications. This idea is supported by the fact that vascular symptoms in SSc result from diffuse microangiopathy, which is a pathological hallmark of the disease, whereas cryoglobulinemic vasculitis arises from immune-complex deposits—mainly mixed cryoglobulins—in small arteries, capillaries, and venules [48].

Some researchers suggest that cryoglobulinemia in SSc patients may be a manifestation of Sjögren’s syndrome associated with SSc [49,50,51]. Interestingly, SSc accompanied by Sjögren’s syndrome tends to be less severe than SSc alone, with a notably lower incidence of pulmonary fibrosis. Furthermore, both conditions often coexist with other autoimmune diseases or autoantibodies, indicating a broader autoimmune tendency possibly linked to a unique genetic profile [51].

Vasculitis can also occur in rheumatoid arthritis (RA). It is important to differentiate between vascular involvement related to RA pathogenesis, isolated digital vasculitis, and clinical rheumatoid vasculitis syndrome. Features such as high rheumatoid factor titers, the presence of cryoglobulins, reduced circulating complement levels, a higher prevalence of HLA-DR4, and characteristic pathological findings all point toward an immune-mediated cause [52].

In patients with rheumatoid arthritis, the presence of cryoglobulins can lead to a specific type of vasculitis, which can cause skin infarction, joint pain, and other systemic symptoms. This can complicate the management of RA, as it may contribute to higher disease activity and more severe symptoms [53].

Cryoglobulins isolated from the serum of patients with RA and SLE were examined for their immunoglobulin content, antibody profiles, and complement components [54]. In both conditions, the cryoglobulins were primarily composed of IgG. IgM rheumatoid factor (RF) was detected in 65% of RA cryoglobulins but was present in only 17% of those from SLE patients. Complement component 1 q (C1q)-binding activity was observed in most RA and SLE serum samples, but within the cryoglobulins, it was detected only in those from SLE patients. RF activity was present in both the serum and cryoglobulins of RA patients. No significant differences were noted in the levels of complement components C3 and C4. The variations in antibody composition and complement-binding capacity between RA and SLE cryoglobulins may influence the characteristics of the immune complexes, including their tendency to deposit in tissues and their potential to cause disease [54].

## 2. Cryofibrinogen

Cryofibrinogen (CF) is a cryoprotein that was first identified in 1955 by Korst and Kratochvil [55]. It is an insoluble complex of fibrin, fibrinogen, fibronectin, factor VIII and other small plasma proteins that quickly forms at cold temperatures and dissolves when warmed to 37 °C [56,57]. Physicochemical mechanisms are not yet fully understood. It seems that cold temperature is responsible for the formation of stabilized CF oligomers; fibronectin probably binds to fibrinogen, and fibrin acts as a nucleus favoring the cold precipitation of CF that can deposit and decrease circulation in small and medium vessels [56].

Another proposed mechanism suggests that elevated plasma levels of protease inhibitors, such as α1-antitrypsin and α2-macroglobulin, along with delayed euglobulin lysis, inhibit plasmin activity and thereby suppress fibrinolysis. This inhibition can lead to the accumulation of cryofibrinogen, which clots in the presence of thrombin, resulting in thrombotic occlusions [58].

The methods used in the laboratory for detecting and measuring CF are critically important. Blood samples must be collected in tubes containing anticoagulants like EDTA or citrate—heparin should be avoided, as it can lead to the spontaneous formation of cryoprecipitating heparin–fibrinogen–fibronectin complexes at 4 °C, potentially causing false-positive results [59,60].

Although cryofibrinogenemia is considered a rare condition, it is likely underdiagnosed. When cryopathy is clinically suspected, cryofibrinogen has been detected in 12% to 51% of patients [61]. It may be asymptomatic, but when symptomatic, it often presents with skin lesions such as ulcers, necrosis, gangrene, and livedo reticularis, as well as joint pain (arthralgia), thrombosis, and limb ischemia [62]. These clinical signs are common in rheumatic diseases, highlighting the importance of a thorough differential diagnosis. Currently, no epidemiological studies have assessed the prevalence of cryofibrinogenemia across various rheumatic conditions [63].

Cryofibrinogenemia can be either primary (idiopathic) or secondary to other conditions such as malignancy, infection, vasculitis, connective tissue diseases, or may coexist with cryoglobulinemia [64]. To date, there are no validated classification criteria for this disorder, and no clear correlation exists between cryofibrinogen levels and disease severity or cold sensitivity [65].

The condition is associated with increased mortality, primarily due to sepsis from gangrene or complications related to the underlying disease [61,66]. Only a few cases of cryofibrinogenemia linked to autoimmune diseases have been documented. One such case involved a 44-year-old woman with cryofibrinogenemia associated with Sjögren’s syndrome, who developed digital necrotic ulcers and purpura on her lower legs [67]. Immunological studies showed a positive rheumatoid factor of 126.9 IU/mL, positive anti-nuclear antibodies with a titer of 1:640, positive anti-SS-A/Ro of >500 U/mL, anti-SS-B/La of 21.3 U/mL antibodies, and anticentromere antibody of 229.2 U/mL.

Cryoprecipitate was identified in the patient’s plasma, and immunoelectrophoresis confirmed it to be cryofibrinogen. Additionally, the patient exhibited symptoms of hyposalivation and sialadenitis, fulfilling the European diagnostic criteria for primary SS [68]. Treatment was initiated with high-dose prednisolone at 60 mg/day, leading to a marked improvement in the ulcerations. Notably, cryofibrinogen, which had been present prior to treatment, was no longer detectable following steroid therapy [67].

An association between systemic sclerosis (SSc) and cryofibrinogenemia has also been reported [69]. However, the clinical, biological, morphological, and prognostic significance of this combination remains unclear. SSc is recognized as a potential secondary cause of cryofibrinogenemia, but the presence of cryoprecipitate does not appear to influence disease phenotype or negatively impact survival outcomes [70].

In a case described by Barrett and colleagues, a 49-year-old man with SSc developed ischemia in all extremities during an especially cold period, which progressed to digital gangrene. Necrotic, sloughing lesions and bullae were observed on the dorsal surfaces of both feet. His plasma cryofibrinogen level measured 435 mg/L, though coagulation studies were within normal limits. Despite treatment with subcutaneous heparin, intravenous Dextran 40, and prostaglandin E1, much of the ischemia was irreversible, and the patient ultimately died. At the time, it was unclear whether cryofibrinogenemia preceded the gangrene, but it is now understood that gangrene can be a clinical manifestation of this condition [69].

Another case involved an 18-year-old Japanese woman who developed cryofibrinogenemia while undergoing methimazole treatment for Graves’ disease, an organ-specific autoimmune condition [71]. She experienced joint pain in both ankles and the right elbow, along with purpura on her lower legs. Initial treatment with acetylsalicylic acid (1.5 mg/day) and prednisolone (15 mg/day) provided some symptom relief. However, symptoms worsened when the prednisolone dose was tapered to 5 mg/day. She subsequently developed livedo reticularis, Raynaud’s phenomenon, and an ulcer on her left big toe. The laboratory data showed a normal coagulation test; the thyroid function test profile was that of euthyroidism; the anti-thyroid-stimulating hormone receptor antibody was positive with a high titer, and the anti-microsomal antibody was also positive; ANA was positive (1:640) with a speckled pattern; lupus anticoagulant (LAC) and anticardiolipin antibody were negative; and cryofibrinogen was positive with a cryocrit of 18%. Her symptoms resolved after administration of prostaglandins and acetylsalicylic acid [71].

Cryofibrinogenemia should be considered when making a diagnosis of cryopathy in a patient with Graves’ disease.

Familial presentation of cryofibrinogenemia has been described [72]. Forty members from a large family with an initial diagnosis of familial cryofibrinogenemia were examined. Seventeen family members reported a history of acrocyanosis with cold exposure. None reported symptoms suggestive of lupus. Exome sequencing identified a heterozygous mutation, D18N, in the TREX1 (three-prime repair exonuclease 1) gene as the underlying cause. TREX1 has a critical role in metabolizing endogenous ssDNA and nucleic acids and preventing their accumulation in the cytosol [73]. In patients with heterozygous mutations, a combination of TREX1 dimers is produced with either normal (TREX1^WT/WT^), decreased (TREX1^WT/D18N^), or fully suppressed (TREX^D18N/D18N^) activity [74]. The resulting intracellular accumulation of ssDNA leads to constant activation of interferon regulatory factor 3 (IRF3), which can lead to a severe auto-inflammatory disorder [75,76].

The mutation is already being associated with autosomal dominant familial chilblain lupus erythematosus (CHLE) [77]. Both entities were found to share highly similar clinical presentations, suggesting they are part of the same syndrome in which cryofibrinogenemia and lupus manifestations have variable penetrance.

It is worth noting that a meta-analysis of 34 genome-wide association studies identified the IRF3 gene loci as a significant determinant of fibrinogen levels, providing further evidence of a relationship between the two pathways [78].

Familial cryofibrinogenemia should be recognized as part of the spectrum of TREX1-related disorders. Reports of cryofibrinogenemia in children are extremely rare [79]. A retrospective analysis of pediatric cases diagnosed with this condition revealed that it can be challenging to identify in children, as the initial symptoms tend to be diverse and non-specific. Common clinical features included muscle weakness, purpura, and joint pain (arthralgia). Laboratory tests showed elevated antithrombin III levels in all patients, while abnormalities in protein S, elevated protein C, low C3, and altered C4 levels were also noted.

Although most of these pediatric cases were classified as secondary cryofibrinogenemia, the prevalence of autoimmune antibodies was relatively low. Interestingly, autoantibodies were often negative at diagnosis but became detectable following treatment, leading researchers to hypothesize that these antibodies may initially be sequestered within vessel walls and only released into circulation after therapy. Therefore, in children presenting with persistent, unexplained pain in multiple areas accompanied by purpura or bruising, cryofibrinogenemia should be considered and further evaluated [79].

Vasculitis associated with RA tends to occur in patients with long-standing disease and severe joint involvement. It is biologically characterized by very high levels of RF and elevated anti-cyclic citrullinated peptide (anti-CCP) antibodies [80].

M.S. Soyfoo and colleagues described a case involving overlapping features of rheumatoid vasculitis and cryofibrinogenemia. The patient, who had a long-standing history of RA, experienced a sudden worsening of symptoms, including fever, bullous skin lesions and ulcers over both ankles, necrotic foot lesions, and mononeuritis. Blood tests revealed extremely high levels of cryofibrinogen, while tests for cryoglobulins, anticardiolipin, and antiphospholipid antibodies were negative. The cryoprecipitate was composed entirely of fibrinogen [81]. Rheumatoid vasculitis is a rare complication of RA, affecting fewer than 5% of patients, and its incidence has significantly declined over time. Nonetheless, the involvement of cryofibrinogenemia in cases of severe cutaneous and digital necrosis warrants further investigation [82].

It is also not unusual for patients to present with both cryofibrinogen and cryoglobulins. In approximately 30% of tested samples, CF positivity was found alongside CG, and this coexistence appeared to be independent of the individual levels of CF and CG.

It is not uncommon to find both CF and CG in patients simultaneously. Positive CFs were associated with CG in 30% of samples, and this association was independent of CF and CG concentrations [64].

The combination of these two cryoproteins is largely due to the coprecipitation of fibrinogen and other proteins within the CG complex. When CG and CF precipitate together, the treatment of CG leads to the CF resolution. Anyway, finding a positive CF without CG is indicative of an alteration of fibrinogen that favors its cold polymerization and the formation of complexes that can hit small vessels [57,83]. The pathogenic role of CF, alone or associated with a CG, remains to be proven. However, they act through different mechanisms. CG involves an immune response, especially through the contribution of Ig with rheumatoid factor activity and complement activation, causing a local inflammatory reaction and vasculitis. On the contrary, cold deposition of CF does not result in an inflammatory reaction but rather in the occlusion of small- and medium-sized vessels.

The clinical outcomes of CF associated with CG or isolated CF or CG are very different [64]. It is important to sensitize clinicians to a systematic and concurrent detection of CF and CG in clinical contexts that are suggestive of the presence of cryoproteins. This can be useful to select treatment, as immunosuppressive agents will be privileged for CG or CG/CF association and fibrinolytic agents for isolated CF [63,64] (Table 2).

## 3. Cold Agglutinins

Autoimmune hemolytic anemias (AIHA) encompass a diverse set of conditions where the body produces autoantibodies that target and destroy red blood cells (RBC). Affecting approximately 0.8–3 per 100,000 adults annually, with a prevalence of 17 per 100,000 and an 11% mortality rate [84], AIHA can arise as a primary disorder (in over 60% of cases) or secondary to conditions like autoimmune diseases, chronic lymphocytic leukemia (CLL), non-Hodgkin lymphoma (NHL), and certain infections (e.g., cytomegalovirus, mycoplasma pneumonia, hepatitis, HIV) [85]. A key factor in classifying AIHA is the temperature at which RBC opsonization and destruction occur, leading to warm, cold, or mixed types [84]. The symptoms of AIHA vary depending on the specific type, commonly including dyspnea, fatigue, headache, muscle weakness, pallor, and jaundice. Notably, cold agglutinin disease (CAD), a specific form of AIHA, can lead to acrocyanosis (discoloration of the extremities upon cold exposure) and Raynaud’s phenomenon, with a rare potential for progression to gangrene [85,86]. Two studies in the United States and Norway (involving 89 and 86 patients, respectively) indicated a typical disease onset between 65 and 67 years. The US study reported acrocyanosis in 44% of patients and Raynaud’s phenomenon in 39%, while the Norwegian study, likely due to the colder environment, observed more severe symptoms in 90% of patients [87,88].

Cold agglutinins (CAs) are produced by B cells at the lymphoplasmacytic cell stage [89], react with antigen at an optimal temperature of 4 °C, but remain active at a varied temperature range.

The pathogenicity of CAs, in addition to the titer defined as an index of CAs activity measured at 4 °C as the inverse of the maximum serum dilution at which agglutination can be observed, depends above all on the thermal amplitude (TA) defined as the highest temperature at which CAs will react with antigen [90]. At a TA > 28–30 °C, red blood cells will agglutinate in the coldest parts of the circulation even at mild room temperatures, undergoing hemolysis. TA can reach the physiological temperature of 37 °C, causing a greater probability of clinically significant manifestations [90].

IgM encoded by the IGVH4-34 gene located on the q arm of chromosome 14 is responsible for over 90% of CA disease cases, and only 7% are cases mediated by IgG and IgA [87]. The agglutinating ability of IgM immunoglobulin is due to its pentameric isotype, which requires only a single antibody to bind a C1 molecule to more efficiently activate the classical complement pathway and cause both intravascular and extravascular hemolysis [91].

In contrast, IgG, being smaller molecules, requires more cold agglutinins; however, IgG3 and IgG1 bind complement much more efficiently than IgG2 and IgG4 [92]. CAs may display affinity for non-ABO blood group carbohydrate antigens present on red blood cell membranes, causing red blood cell agglutination and subsequent hemolysis [93]. Additionally, 90% of anti-ICAs are expressed on adult RBC membranes and are more pathogenic than those with anti-i specificity expressed on predominantly fetal and neonatal RBCs up to 18 months after birth [87]. The recognition of antigen I mediated by the Framework 1 (FR1) region of the heavy variable region of IgM and the binding specificity of the light chain are crucial to understanding the variations in the thermal amplitude of CA and consequently the clinical phenotype. Clinically, anti-I antibodies may be associated with Mycoplasma pneumoniae infections, while anti-i antibodies may be associated with Epstein–Barr virus mononucleosis in adolescents or young adults [84]. Although most of the defining criteria for CAD do not require the presence of anti-I/anti-I specificity, a group of authors include it as a criterion for diagnosis [84]. Conflicting data regarding the hemolysis of cold agglutinins of the IgM isotype have been reported by researcher Sniecinski and his collaborators [94]. In fact, RBC agglutination due to high titers of anti-I-type CA in COVID-19 patients without hemolytic anemia did not show hemolysis, as is also known in Mycoplasma pneumoniae [95].

Nowadays, cold-reactive autoantibodies cause cold agglutinin disease (CAD), cold agglutinin syndrome (CAS) and paroxysmal cold hemoglobinuria (PCH). The first two pathological conditions represent 15–25% of AIHA with an incidence of 1 per million people/year [85,87,96], while PCH is among the rarest forms of AIHA, with an estimated annual incidence of 0.04 cases per 100,000 people [97].

The World Health Organization (WHO) and the International Consensus Classification (ICC) have recognized cold agglutinin disease (CAD) as a new diagnostic entity distinct from cold agglutinin syndrome (CAS). CAD is a primary, chronic condition in which the lymphoproliferation of a B-cell clone produces a cold-reactive (≤30 °C) IgM monoclonal autoantibody, and CAS is a secondary condition often associated with infection or malignancy [98].

The AIHA international consensus document defines CAD as an AIHA characterized by positivity for the complement fragment C3d and a CA titer of 64 or higher at 4 °C and negativity for IgG that may be weakly positive in 20% of patients [99]. It has been documented that bone marrow biopsy of patients with primary CAD frequently shows a subtype of malignant lymphoma, lymphoplasmacytic lymphoma and more rarely diffuse large B-cell lymphoma (DLBCL), although it is very rare. Laboratory tests revealed anemia and elevated bilirubin and cold agglutinins with a titer of 8192 at 4 °C and <1 at 37 °C [100]

In cases of adult populations with a clonal B-cell lymphoproliferative disorder (LPD) in blood or bone marrow without hemolysis and clinical evidence of malignancy, CA titers <64 have been identified as polyclonal, whereas higher titers are usually associated with severe anemia dependent on the antibody isotype, thermal amplitude, degree of complement activation, titer, and specificity of the cold agglutinin [84].

It has been reported in the literature that in CAD the monoclonality of CA is almost entirely related to IgM light chains (IgM κ), 7% and 5% to IgM light chains (IgM λ) and IgG, respectively, and rare cases involve IgA [101,102].

CAS is a transient secondary disorder that occurs following a bacterial or viral infection due to mycoplasma pneumoniae, Epstein–Barr virus, adenovirus, CMV, influenza viruses, varicella zoster virus, human immunodeficiency virus, Escherichia coli, Listeria monocytogenes, and Treponema pallidum, malignancies, and autoimmune disorders [103]. Some studies link CAS to Severe Acute Respiratory Syndrome Coronavirus 2 (SARS-CoV-2) [104]. Unlike CAS secondary to aggressive B-cell lymphoma, which may be characterized by a monoclonal IgMλ or IgMκ component, in CAS secondary to Mycoplasma infection, the high-titer specific anti-I IgM-CA derived from lymphoplasmacytic cells are polyclonal. Hemolytic anemia following pneumonia is rather sudden in the second week of Mycoplasma infection and usually resolves within 4–6 weeks [105].

CAS associated with lymphoproliferative disorders (e.g., CLL, lymphomas, and Waldenstrom’s macroglobulinemia) has a chronic course.

Immunoglobulins IgM play a crucial role in the pathogenesis of 90% of cases; rare are cases mediated by IgG and IgA [99]. Through colder extremities (28–30 °C) of the body, IgM is activated, which binds with the Fc portion of the complement protein C1. The Ig-C1 complex transiently bound to the surface of the red blood cells continues through the warmer areas of the body, activating the C1q esterase and the proteins C4 and C2 and the C3 convertase, which produces C3a and C3b [96].

The red blood cells coated with C3b are sequestered and destroyed by macrophages in the reticuloendothelial system, particularly by Kupffer cells in the liver, causing extravascular hemolysis. However, C3b on cells spared from phagocytosis is cleaved into iC3b (inactive), C3c and C3d, which in patients with CAD occupies the potential binding sites for C4 and C3, preventing hemolysis of the patient’s own red blood cells. Furthermore, activation of the complement cascade continues with the binding of C4bC2a to C3b and activation of the enzyme C5 convertase [106,107], resulting in the formation of C5a, a potent anaphylatoxin and C5b, which remains bound to the cell, contributing to the generation of the membrane attack complex (MAC) with proteins C6, C7, C8 and C9 triggering intravascular hemolysis [108].

Paroxysmal cold hemoglobinuria (PCH), discovered in 1904 by Julius Donath and Karl Landsteiner, is defined as a rare autoimmune hemolytic anemia constituting 1–5% of AIHA caused by IgG autoantibodies, in particular the Donath–Landsteiner autoantibody, a bithermic hemolysin that binds to the anti-P antigen of red blood cells at temperatures below 37 °C, causing their destruction and activation of complement [109,110,111]. Thermal amplitude is usually less than 20 °C [112]. PCH may occur as a transient condition in neonates after a viral infection or as a chronic condition in adults with blood cancers or advanced syphilis [96,113,114]. When the red blood cells are warmed to body temperature, the antibody activates complement, and the IgG antibody subsequently dissociates from the red blood cells [115]. Opsonization of RBCs in AIHA with IgG antibodies occurs in the spleen, whereas IgG plus complement or complement alone on RBCs occurs in the liver, particularly in Kupffer cells [116]. In contrast to cold agglutinin disease, where hemolysis is extravascular due to C3b, in paroxysmal cold hemoglobinuria, activation of the membrane attack complex causes cell lysis on rewarming (biphasic antibody). Intravascular hemolysis of PCH leads to dark urine, flank pain, fever and chills [112].

In CAD, there is a high proportion of coexistent cryoglobulinemia. It has been recently reported that in a cohort of 134 patients with type I cryoglobulinemia concurrent CAD was found in 15% [117]. Critical ischemia and gangrene were seen exclusively in those with cryoglobulinemia and CAD together. This suggests the addition of cryoglobulinemia affects the clinical phenotype of CAD [118].

Both cryoglobulinemia and CAD may be considered monoclonal gammopathies of thrombotic significance due to intravascular occlusion from type I cryoglobulins or complement-activated hemolysis in CAD.

All patients with CAD should be screened for cryoglobulins, particularly if they have circulatory symptoms.

CAD is significantly associated with the risk of venous thromboembolism (VTE), as highlighted in the literature [119]. A strong positive direct Coombs test result for IgG1 and IgG3 confirmed the diagnosis of warm antibody-induced autoimmune hemolytic anemia (wAIHA) and supported the development of pulmonary embolism (PE) [120].

A retrospective study demonstrated that TVE was more common in patients with wAIHA than in patients with CAD and that hemolytic parameters were similar in patients with and without TVE, suggesting that hemolysis was not the only discriminating factor and that, although complement activation was common, it was not a reliable predictor of thromboembolism [121]. Therefore, in agreement with the previous study, anticoagulant prophylaxis should be considered at the time of HAE diagnosis, especially during phases of intense hemolysis, regardless of the presence of triggering factors [121].

## 4. Cryoproteins Assessment

Cryoprotein testing requires the simultaneous collection of serum and plasma to detect both CGs and CF [122]. Since CF is a part of cryoproteins, its analysis cannot be separated from CGs. The concurrent testing of CG and CF displays four events: presence of a precipitate only in serum; presence of a precipitate only in plasma; presence of a precipitate in both serum and plasma; and absence of precipitate.

CG samples are collected in 10 mL serum tubes (no additives), while CF samples are collected in 10 mL K2-EDTA tubes. Heparin is not used to avoid interference. To prevent premature precipitation, all samples are kept at 37 °C during collection and transport. Following clotting, centrifugation is performed at 37 °C (1500× *g* for 15 min), and plasma/serum is stored at 4 °C for a maximum of seven days. Cryoprecipitate (CPT) formation is verified by warming one tube to 37 °C for dissolution. Lipemic, hemolyzed, or icteric samples are discarded.

The analytical phase starts with a visual comparison of CPT formation in serum and plasma at 4 °C. CF is confirmed if CPT is exclusively present in plasma. If CPT is present in both but differs in quantity, CF levels are estimated based on the plasma–serum difference. Cryocrit is quantified by centrifuging a sample in a Wintrobe tube at 500× *g* for 15 min, with a detection limit of 1%.

For characterization, CPT undergoes washing and centrifugation (three cycles at 4 °C, 1500× *g*, 15 min) to remove residual plasma/serum. Dissolution occurs by adding preheated physiological solution at 37 °C. Immunofixation electrophoresis (IFE) is then performed. CG typing uses antisera against IgG, IgA, IgM, kappa, and lambda, following Brouet’s classification [9]. CF detection relies on anti-fibrinogen antiserum, ensuring electrophoresis control with a fixative lane to confirm complete CPT washing. If an albumin band appears, the sample is considered contaminated.

In the post-analytical phase, IFE confirms the presence of CF and/or CGs based on precipitation patterns in respective lanes. Reports indicate CF and CGs as positive/negative, with cryocrit reported as a percentage or <1% if below detection limits. CG-positive results specify immunoglobulin class and type (I, II, III). If CF testing is ordered alone and CGs are found, clinicians are advised to request a separate CGs-specific test.

Current methods for detecting cryoproteins, largely unchanged for decades and involving multiple steps, suffer from inherent challenges and a lack of standardized pre-analytical procedures. Traditional techniques like electrophoresis and immunofixation, used for cryoprotein characterization, cannot identify and track specific antibody lineages (clonotypes) that can evolve and alter their disease-causing potential over time. To address these limitations, Lee and colleagues [123] introduced a novel bedside-to-bench proteomic approach using mass spectrometry. In a study of primary Sjögren’s syndrome patients with mixed cryoglobulinemia, they successfully identified the immunoglobulin heavy chain variable region (IGHV) subfamilies and the patterns of mutations in their cryoprecipitable IgM rheumatoid factor (IgM-RF). This detailed analysis of the heavy chain sequence allowed for the classification of cryoglobulins based on their unique molecular “barcodes” and can be further applied to characterize their light chain components, requiring only 0.2 μg of cryoprecipitate. This advancement opens avenues for developing personalized therapies targeting specific antibody lineages (clonotypes) [123].

In the presence of cold agglutinins, the complete blood count (CBC) shows falsely low red blood cell count and hematocrit values, with a relative increase in mean corpuscular volume (MCV) due to reticulocytosis and agglutinated red blood cells [103].

Peripheral blood smear confirms agglutination, and due to the loss of membrane from the antibody-coated red cells, spherocytes are common [112]. For the normalization of the above parameters, determination of the blood count is performed by placing the peripheral blood sample in incubation at 37 °C for one hour. Biochemical markers secondary to hemolysis include indirect bilirubin and lactate dehydrogenase (LDH) resulting in slight elevation, while haptoglobin is reduced [84].

## 5. Conclusive Remarks

In recent years the co-occurrence of CGs and autoantibodies in the course of HCV infection and autoimmune syndromes has been a matter of intense research and debate. B cell stimulation due to the HCV can result in autoimmunity, with rising levels of CGs, RF and free light chains of immunoglobulins associated with a wide range of cryoglobulinemic symptoms [124]. But the relationship between cryoproteins and non-HCV autoimmune diseases has not been fully elucidated, maybe because they are in themselves quite rare or underdiagnosed events.

Less is known about cryofibrinogenemia if compared with cryoglobulinemia, even if they occur in autoimmune disease (Table 2). Methods for CG and CF detection and quantification are downright crucial, and the concurrent detection is pivotal for the diagnosis. More than 90% of cases of cryoglobulinemia have a known underlying cause; therefore, treatment is focused on the cause of the disorder rather than merely symptomatic relief [125].

Cold agglutinins are autoantibodies that target red blood cell antigens at temperatures below mean core body temperature. They produce RBC agglutination and cold agglutinin disease, a rare form of autoimmune hemolytic anemia. Due to its under recognition, there is a delay between the start of symptoms and the diagnosis. Therapeutic approaches have been targeted against the clonal lymphoproliferation in CAD or the underlying disease in CAS [90]. Non-pharmacological management consists of thermal protection.

Further research should investigate whether the cold agglutinin and cryoglobulin represent properties of the same monoclonal protein and explore the influence of cryoglobulins on CAD.

## Figures and Tables

**Table 1:** Classification of CGs. Table 1 shows the different compositions of CGs according to single or mixed components and to their mono- or polyclonality, as described by Brouet et al. [9], and their occurrence in associations to infective/autoimmune/lymphoproliferative disorders.

**Cryoglobulinemia Type I**			
Immunoglobulin classes	Associated disorders	Symptoms	Percent of cases
**Monoclonal Ig** IgM (mainly) IgG (IgG2 or IgG3) IgA (rarely)	Lymphoproliferative disorders	Vasculitis or distal gangrene/necrosis	10–15%
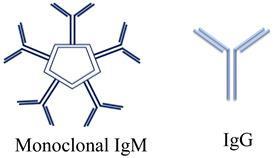			
**Mixed Cryoglobulinemia Type II**			
Immunoglobulin classes	Associated disorders	Symptoms	Percent of cases
**One or more monoclonal Ig (RF activity) + polyclonal Ig** IgM vs. IgG IgG vs. IgG	Infective disorders (HCV, HBV, HIV), autoimmune disorders, lymphoproliferative disorders, chronic liver diseases	Purpura, arthralgia, Raynaud’s phenomena	65%
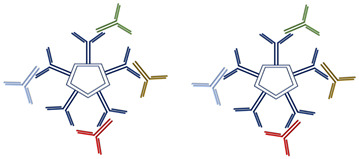			
**Mixed Cryoglobulinemia Type III**			
Immunoglobulin classes	Associated disorders	Symptoms	Percent of cases
**Polyclonal Ig or oligoclonal Ig** **(RF activity) + polyclonal Ig (microeterogeneous)** IgG-IgM IgM-IgG-IgA	Infective disorders (HCV, HBV, HIV), autoimmune disorders, lymphoproliferative disorders	Purpura, arthralgia, Raynaud’s phenomena	25%
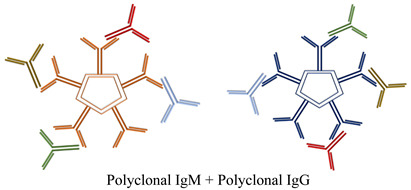			

**Table 2:** Cryoglobulins and cryofibrinogen involvement in autoimmune diseases.

**Cryoglobulins and Induced-Autoimmune Vasculitis**	
**Autoimmune Disorders**	**Reference**
Autoimmune hepatitis	Jeyapraniya A et al. [14] Juran BD et al. [17] Evans JT et al. [19]
Coexistence of celiac disease with autoimmune hepatitis	Biecker E et al. [18]
Sjögren’s disease	Argyropoulou OD et al. [22] Argyropoulou OD et al. [23] Mavragani CP et al. [24] De Vita S et al. [27]
Systemic lupus erythematous	Karimifar M et al. [34] Winfield JB et al. [36] Gripenberg M et al. [37] Adu D et al. [38] Zvaifler NJ et al. [39]
Systemic sclerosis (SSc)	Buskila D et al. [40] Husson JM et al. [41] Invernizzi F et al. [42] Deguchi A et al. [47] Quéméneur T et al. [49]
Rheumatoid arthritis	Vollertsen RS et al. [52] Suszek D et al. [53] Erhardt CC et al. [54]
**Cryofibrinogen and Autoimmunity**	
**Autoimmune Disorders**	**Reference**
Sjögren’s syndrome	Yoshida K et al. [67] Vitali C et al. [68]
Systemic sclerosis	Barrett MC et al. [69]
Graves’ disease	Hosoi K et al. [71]
Rheumatoid arthritis (RA)	Genta MS et al. [80]
